# Arl13b influences cilia length in the zebrafish Kupffer’s vesicle

**DOI:** 10.1186/2046-2530-1-S1-P110

**Published:** 2013-04-26

**Authors:** P Pintado, D Barral, SS Lopes

**Affiliations:** 1 Faculty of Medical Sciences, CEDOC

## 

Joubert Syndrome is a ciliopathy that can be caused by a mutation in Arl13b protein. This syndrome is characterized by problems in embryonic development, especially at the neurological level. Arl13b is a protein that belongs to the small GTPase family, but presents the double size of a normal GTPase, because it has a different C-terminus with a coiled-coil domain and proline rich region. It is known that Arl13b localizes to the cilium and recent data showed that Arl13b is in the ciliary membrane. However the molecular function of Arl13b is unknown. This work is based on a functional study of this protein where we used zebrafish as a vertebrate model organism to study the embryonic development in a situation of loss or gain of function of Arl13b. In both situations we observed cardiac edema and abnormal body curvature. This work shows that in an over-expression situation cilia length is increased in the Kupffer’s vesicle, leading to randomization of the left-right gene expression cascade. This study contributes to a better understanding of Arl13b protein function in an organism since its molecular mechanism is not yet known, and provides new information on the localization of this protein in motile cilia.

### Publisher's note

Due to an administrative error this abstract was omitted from the original supplement submission and thus the publication was updated on 26 April 2013.

